# The ability to accumulate deoxyuridine triphosphate and cellular response to thymidylate synthase (TS) inhibition

**DOI:** 10.1054/bjoc.2001.1921

**Published:** 2001-08

**Authors:** S D Webley, S J Welsh, A L Jackman, G W Aherne

**Affiliations:** CRC Centre for Cancer Therapeutics, Institute of Cancer Research, 15 Cotswold Road, Sutton, Surrey, SM2 5NG

**Keywords:** dUTPase, thymidylate synthase, ZD9331, dUTP, uracil misincorporation

## Abstract

Thymidylate synthase (TS) is an important enzyme catalysing the reductive methylation of dUMP to dTMP that is further metabolized to dTTP for DNA synthesis. Loss of viability following TS inhibition occurs as a consequence of depleted dTTP pools and at least in some cell lines, accumulation of dUTP and subsequent misincorporation of uracil into DNA. The expansion in dUTP pools is largely determined by the expression of the pyrophosphatase, dUTPase. Our previous work has shown that following TS inhibition the ability to accumulate dUTP was associated with an earlier growth inhibitory effect. 3 human lung tumour cell lines and HT29 human colon tumour cells transfected with dUTPase have been used to investigate the relationship between loss of viability following TS inhibition and dUTP accumulation. Cell cycle arrest typical of TS inhibition was an early event in all cell lines and occurred irrespective of the ability to accumulate dUTP or p53 function. However, a large expansion of dUTP pools was associated with mature DNA damage (4 h) and an earlier loss of viability following TS inhibition compared to cells in which dUTP pools were not expanded. In A549 cells damage to mature DNA may have been exacerbated by significantly higher activity of the excision repair enzyme, uracil-DNA glycosylase. Consistent with results using different inhibitors of TS, transfection of dUTPase into HT29 cells significantly reduced the cytotoxicity of a 24 h but not 48 h exposure to ZD9331. Although loss of viability can be mediated through dTTP deprivation alone, the uracil misincorporation pathway resulted in an earlier commitment to cell death. The relevance of this latter pathway in the clinical response to TS inhibitors deserves further investigation. © 2001 Cancer Research Campaign http://www.bjcancer.com


*British Journal of Cancer* (2001) **85**(3), 446–452
© 2001 Cancer Research Campaign
doi: 10.1054/ bjoc.2001.1921, available online at http://www.idealibrary.com on IDEAL®

http://www.bjcancer.com

### The ability to accumulate deoxyuridine triphosphate and cellular response to thymidylate synthase (TS) inhibition

**SD Webley, SJ Welsh, AL Jackman and GW Aherne**

CRC Centre for Cancer Therapeutics, Institute of Cancer Research, 15 Cotswold Road, Sutton, Surrey, SM2 5NG

**Summary** Thymidylate synthase (TS) is an important enzyme catalysing the reductive methylation of dUMP to dTMP that is further metabolized to dTTP for DNA synthesis. Loss of viability following TS inhibition occurs as a consequence of depleted dTTP pools and at least in some cell lines, accumulation of dUTP and subsequent misincorporation of uracil into DNA. The expansion in dUTP pools is largely determined by the expression of the pyrophosphatase, dUTPase. Our previous work has shown that following TS inhibition the ability to accumulate dUTP was associated with an earlier growth inhibitory effect. 3 human lung tumour cell lines and HT29 human colon tumour cells transfected with dUTPase have been used to investigate the relationship between loss of viability following TS inhibition and dUTP accumulation. Cell cycle arrest typical of TS inhibition was an early event in all cell lines and occurred irrespective of the ability to accumulate dUTP or p53 function. However, a large expansion of dUTP pools was associated with mature DNA damage (4 h) and an earlier loss of viability following TS inhibition compared to cells in which dUTP pools were not expanded. In A549 cells damage to mature DNA may have been exacerbated by significantly higher activity of the excision repair enzyme, uracil-DNA glycosylase. Consistent with results using different inhibitors of TS, transfection of dUTPase into HT29 cells significantly reduced the cytotoxicity of a 24 h but not 48 h exposure to ZD9331. Although loss of viability can be mediated through dTTP deprivation alone, the uracil misincorporation pathway resulted in an earlier commitment to cell death. The relevance of this latter pathway in the clinical response to TS inhibitors deserves further investigation. © 2001 Cancer Research Campaign http://www.bjcancer.com

**Keywords**: dUTPase; thymidylate synthase; ZD9331; dUTP; uracil misincorporation

5-Fluorouracil (5-FU) has been used for some time for the treatment of malignant disease and mechanism by which the drug is thought to act is through inhibition of thymidylate synthase (TS). In addition, several antifolate TS inhibitors have recently been investigated for their clinical use as chemotherapeutic agents (Jackman and Judson, 1996). TS is a rate-limiting enzyme catalysing the reductive methylation of dUMP to dTMP which is further metabolized to dTTP for DNA synthesis. Inhibition of TS results in depletion of intracellular dTTP pools and an elevation in dUMP pools (Jackson et al, 1983; Aherne et al, 1996). In some cell lines (but not all) expanded dUTP pools are also a consequence of TS inhibition.

It is not entirely clear how dNTP perturbations following TS inhibition result in cell death, but this is often assumed to occur through uracil misincorporation. When significantly elevated, dUTP can be misincorporated into DNA in place of dTTP by DNA polymerase (Brynolf et al, 1978). Incorrectly inserted dUTP coupled with the action of uracil-DNA glycosylase (UDG) results in a futile cycle of misrepair leading eventually to DNA strand breaks and cell death (Goulian et al, 1986). Although this concept is widely supported by several studies (reviewed in Aherne and Brown, 1998), dUTP accumulation may not be a critical event accompanying TS inhibition in all cells. dTTP deprivation itself, if it occurs for a long enough period of time (greater than a generation time), induces cell death (Houghton et al, 1994; Matsui et al, 1996). dATP has also been reported to be elevated following TS inhibition and it has been proposed that this triggers cellular events which result in the cleavage of DNA (Chong and Tattersall, 1995; Houghton et al, 1995; Sundseth et al, 1997).

dNTP perturbations during TS inhibition result in DNA damage (Curtin et al, 1991; Canman et al, 1994; Matsui et al, 1996), the response to which may essentially be governed by key downstream proteins such as the tumour suppressor p53. Activation of p53 results in the transactivation of several proteins involved in G1 cell cycle arrest, apoptosis and DNA repair (O'Connor, 1987; Zambetti and Levine, 1993). The specific TS inhibitor raltitrexed (RTX; Tomudex™; ZD1694) has been shown to induce DNA damage at both the single- and double-stranded level (Yin et al, 1992; Matsui et al, 1996) and p53 has been reported to be responsive to the effects of this drug (Arrendondo et al, 1994). Another extensively studied function of p53 is as a G1 cell cycle checkpoint, where the protein is elevated in response to DNA damage (Hartwell and Kastan 1994; Nelson and Kastan, 1994). Cell cycle alterations typical of G1/S phase arrest appear to be a universal consequence of TS inhibition (Matsui et al, 1996; Tonkinson et al, 1996; Skelton et al, 1998).

The purpose of this study was to further investigate the importance of uracil misincorporation in cell death following inhibition of TS. ZD9331 (Jackman et al, 1997) is a specific non-polyglutamatable antifolate TS inhibitor which is currently being clinically evaluated. Unlike other TS inhibitors this compound does not require metabolic activation and the duration of TS inhibition can be more readily controlled. Earlier studies using this agent have shown that TS activity or protein expression, TS inhi-

*Received 25 October 2000*
*Revised 24 April 2001*
*Accepted 26 April 2001*

*Correspondence to:* GW Aherne bition as determined by dTTP depletion or intracellular ZD9331 levels could not account for differences in sensitivity between 4 human lung tumour cell lines (Webley et al, 2000). A significant difference in the extent of dUTP accumulation following ZD9331 was observed. For example in the A549 cell line, increased pools of dUTP was an early event and over 24 h considerable amounts of dUTP accumulated. In contrast, in MOR cells dUTP pools were only marginally increased over those of untreated cells, even when the dose and duration of drug exposure were increased several-fold. dUTP accumulation was negatively associated with the expression of dUTPase, the pyrophosphatase responsible for maintaining low intracellular levels of dUTP. These results were consistent with reports on the role of dUTPase in mediating the effects of TS inhibition (Canman et al, 1993, 1994). In the current study, the relative roles of dUTP accumulation and dTTP depletion in cell cycle arrest and cell death following exposure of cells to ZD9331 in 3 of these cell lines has been addressed. The study has also briefly assessed the roles of UDG and p53 in downstream events following TS inhibition. The mutant p53 HT29 human colon carcinoma cell line transfected with *E. coli* dUTPase or vector control (Canman et al, 1994) were used as a paired isogenic model.

### MATERIALS AND METHODS

### Materials

All standard laboratory chemicals used in this study were of AnalaR® grade purchased either from British Drug Houses (BDH) (Poole, Dorset UK) or from Sigma (Poole, Dorset UK). ZD9331 was synthesized at Zeneca Pharmaceuticals (Macclesfield, Cheshire, UK), dissolved in 0.15 M sodium bicarbonate ($NaHCO_3$) (pH 8.3) and filter sterilized. [$^{14}$C]-thymidine (specific activity 51.4 Ci $mMol^{-1}$) and [methyl-$^3$H] thymidine (specific activity 5.0 Ci $mMol^{-1}$) were purchased from Amershan International plc.

### Cell culture

The human lung carcinoma cell lines, A549 (squamous), MOR (adenocarcinoma) and HX147 (large cell) were maintained at 37 ˚C in air containing 5% $CO_2$ in DMEM tissue culture medium (Gibco, Paisley, Scotland) and 10% heat inactivated dialysed fetal bovine serum (FBS) supplemented with 2 mM glutamine, 50 μg $ml^{-1}$ gentamycin, 2.5 μg $ml^{-1}$ fungizone, 10 μg $ml^{-1}$ insulin and 0.5 μg $ml^{-1}$ hydrocortisone. The human HT29 colon adenocarcinoma *E. coli* dUTPase transfected cell line dutE7 and its neotransfected control CON were kindly provided by Dr Jonathan Maybaum. These cells were maintained using the above conditions. All cells were free of mycoplasma during experimentation. Growth inhibition was measured using MTT assay as previously described (Webley et al, 2000).

### Flow cytometry

Following ZD9331 treatment (1 μM for 4 or 24 h), adherent cells ($1 \times 10^6$) were harvested by gentle scraping in 10 ml of ice cold PBS and pelleted by refrigerated centrifugation for 5 min at 2500 rpm (600 ***g***). Cell pellets were resuspended in 200 μl PBS and 2 ml of ice-cold 70% ethanol was added to the cell suspension (whilst being vortexed) and left for at least 30 min at 4˚C. The cells were centrifuged at 2500 rpm (600 ***g***) for 5 min (4˚C), resuspended in 800 μl of PBS into which 100 μl RNase (1 mg $ml^{-1}$) and 100 μl propidium iodide (400 μg $ml^{-1}$) were added. After a 30 min incubation period at 37˚C, cell cycle analysis was performed using an argon-ion laser (Coulter Ltd, Luton UK) operated at 488 nm. Following pulse shape analysis and gating on a cytogram of orthogonal *vs* forward light scatter, a DNA histogram of cell number against red (DNA-PI) fluorescence was recorded.

### Colony-forming assays

Cells were plated in a T-25 tissue culture flask ($2 \times 10^5$ cells/5 ml) and exposed to ZD9331 1 μM) once exponential growth had been reached. After 4, 24 or 48 h drug exposure, cells were harvested using cell dissociation solution (Sigma) and suspended in 8.5 ml of culture medium containing non-dialysed FBS. After centrifugation at 1500 rpm (300 ***g***) for 5 min (room temperature), cells were resuspended in 2 ml of the same medium and then diluted to between 400–1000 cells/10 ml and plated onto 10 cm Petri dishes. The cells were left to form colonies for 14 days, after which the colonies were stained with 0.2% crystal violet in PBS containing 10% formalin and those with greater than 50 cells counted. The average plating efficiency for each cell line was approximately 40%. The loss of colony formation was expressed as the percentage plating efficiency of the test cells compared to the plating efficiency of the control cells.

### Measurement of DNA damage by alkaline elution

The method used to measure DNA single-strand breaks following TS inhibition has been described previously (Curtin et al, 1991). Irradiated internal standard cells were co-eluted with experimental cells to allow standardization of elution rate. For studies measuring mature DNA damage, experimental cells were incubated with 0.4 μCi [$^{14}$C] Thd and internal standards were incubated with 1 μCi [methyl-$^3$H] Thd for 24 h. After a 4–24 h post-labelling chase period in fresh medium, the medium was replaced with ZD9331-containing medium for 4, 24 or 48 h. For measurements of nascent DNA damage, radiolabelled Thd was present for only the last 4 h of drug treatment prior to harvesting. For quantitation, the retention of sample cell DNA ($^{14}$C) was evaluated at the point when the retention of internal standard ($^3$H) was 0.5. The rad equivalents were determined in each experiment as previously described (Kohn, 1981).

### UDG assay

$5 \times 10^5$ cells were lysed (50 mM Tris-HCl (pH 7.4), 0.25 M NaCl, 0.1% NP40, 50 mM NaF and 5 mM EDTA) frozen at –80˚C, thawed, and sonicated twice for 30 s (10 amplitude microns, Soniprep 150 MSE). Following centrifugation at 9000 ***g*** for 10 min at 4˚C the supernatant was used to determine UDG activity. The UDG assay (Caradonna and Cheng, 1980) is based on the release of $^3$H-uracil from a [$^3$H] uracil-containing calf thymus DNA preparation that was kindly prepared by Dr Salvatore Caradonna. The assay was performed using the procedures described previously (Muller-Weeks and Caradonna, 1996). Briefly, reactions took place at 37˚C in a volume of 100 μl containing 45 μl 100 mM Tris HCI pH 7.5, 4 mM DTT, 10 mM EDTA, 0.2 mg $ml^{-1}$ BSA, 2 μl calf thymus [$^3$H]uracil-DNA substrate (8.35 nmoles $ml^{-1}$) and 3 μl water. The reaction was

© 2001 Cancer Research Campaign 

initiated by the addition of 50 μl of diluted cell extract and left for 5 to 10 min. 25 μl of salmon sperm (DNA 1 mg $ml^{-1}$) (Sigma) and 25 μl 4M PCA were used to terminate the reaction. After 10 min on ice, the samples were centrifuged at 18 000 rpm (9000 ***g***) in a Micro Centaur (MSE) centrifuge for 10 min and the amount of radioactivity in the supernatant counted. A unit of UDG activity was expressed as the amount of glycosylase required to release 1 pmol of uracil $min^{-1}$ $mg^{-1}$ of protein at 37˚C.

### Western immunoblot analysis

Following exposure of $1 \times 10^6$ cells to 1 μM ZD9331 for 4, 24 or 48 h, Western blot analysis was performed according to standard protocol, and the protein bands of interest were probed using either a mouse monoclonal antibody to p53 (D01) (Autogen Bioclear, UK, Calne, Wiltshire) or p21 (Santa Cruz, clone 19). The expression of tubulin was also determined using a mouse monoclonal anti-α-tubulin (Clone B-5-1-2, Sigma) to provide evidence of equal protein loading. In irradiation studies, cells were exposed to 5 Gy delivered using a $^{137}Cs$ source-to-flask distance of 40 cm and a dose rate of 1.36 Gy $min^{-1}$ then left for 4 h before harvesting. Visualization of the protein bands of interest was performed using enhanced chemiluminescence (ECL) reagents (Amersham International).

### Protein estimation

Protein concentrations were measured in cell lysates using the BCA assay (Perbio) according to the manufacturer's instructions.

### RESULTS

### Changes in the cell cycle following ZD9331

Representative DNA histograms generated following ZD9331 (1 μM) treatment of A549, HX147 and MOR cells for 4 and 24 h are shown in Figure 1. Prior to ZD9331 exposure, all cell lines showed a cell cycle profile typical of cells in log phase growth, with most cells in G1 and the remaining cells distributed between S and G2/M. A 4 h exposure to ZD9331 resulted in the disappearance of a discernible G2/M peak in all cell lines. A longer drug exposure (24 h) caused an increase in the percentage of cells in G1 and a decrease in the percentage of cells in S phase. In A549 cells, there was evidence of a sub G1 peak that may represent cell debris following a 24 h exposure to ZD9331. By 48 h, this peak had broadened and a sub-G1 peak was clearly discernible (data not shown). Profiles of this type result from the presence of apoptotic cells (Darzynkiewicz et al, 1992).

### Reversal of ZD9331 cell cycle effects following re-suspension in DFM

The HT29 dutE7 and CON cell lines showed similar cell cycle profiles following a 24 h exposure to 1 μM ZD9331 i.e. loss in the G2/M peak (Figure 1). To establish whether the drug-treated cells retain their ability to synthesize DNA and re-enter the G2/M phase once extracellular drug is removed, dutE7 and CON cells were treated with 1 μM ZD9331 (24 h) and then resuspended in non-dialysed DFM for a further 24 h before cell cycle analysis. Once the drug was removed from the medium, the G2/M peak in both cell lines re-

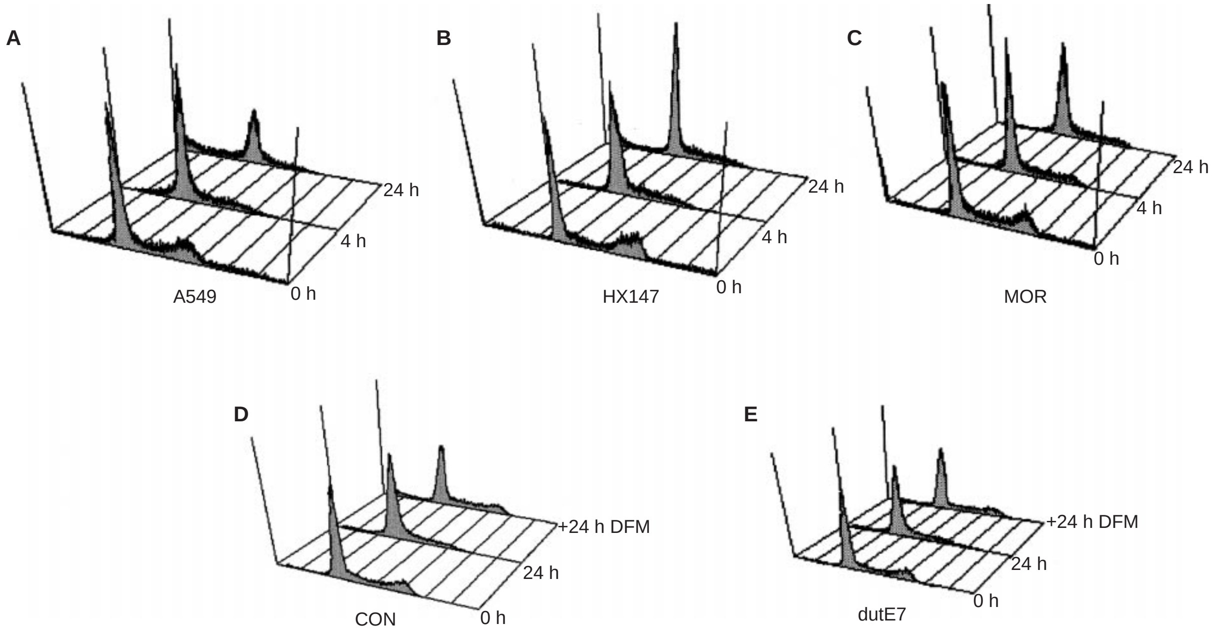

**Figure 1** DNA histograms of cell cycle changes following 1 μM ZD9331 in A549 (**A**), HX147 (**B**) and MOR (**C**) cells at 0, 4 and 24 h after ZD9331 (1 μM) and in CON (**D**) and dutE7 (**E**) cells 0 h and 24 h after ZD9331 (1 μM) and after a further 24 h period in drug-free medium (DFM). At the indicated times after dosing, cells were fixed with 70% ethanol and stained with propidium iodide as described in the Materials and Methods. Histograms are representative of the results of 2 experiments

© 2001 Cancer Research Campaign emerged indicating that some cells at this point were capable of leaving S phase and could re-enter the G2/M phase of the cell cycle.

### ZD9331 induced cytotoxicity

The 24 h growth inhibitory $IC_{50}$ concentrations previously determined by MTT assay for these cell lines were 0.11, 0.17 and 0.28 µM for A549, HX147 and MOR respectively (Webley et al, 2000). A 4 h exposure to ZD9331 (1 µM) had no effect on viability in any of the cell lines. However, by 24 h, A549 cells showed a significant ($P < 0.0001$) loss of viability (7.7% of control) which was further reduced to 0.96% of control after 48 h in drug (Figure 2). The viability of HX147 cells was also significantly reduced to 36.5 and 2.9% survival at 24 h ($P = 0.01$) and 48 h ($P < 0.001$) respectively. In contrast, MOR cells were more resistant to the cytotoxic effect of ZD9331 at 24 h compared to both HX147 and A549 cells, and did not begin to significantly lose the ability to form colonies until 24–48 h in drug ($P < 0.0001$). Indeed MOR cells were significantly ($P < 0.05$) more resistant to a 24 h exposure to ZD9331 than A549 cells.

The 24 h growth inhibition $IC_{50}$ concentrations determined by MTT assay for CON and dutE7 were 0.22 µM ± 0.05 and 15.6 µM ± 3 respectively. The 120 h $IC_{50}$ values were 0.04 µM ± 0.01 and 0.152 µM ± 0.04 for CON and dutE7 respectively (Brown et al, 1997). By 24 h ZD9331 was significantly more cytotoxic in the neotransfected controls than in the dUTPase transfected cells (Figure 3). By 48 h however both cell lines showed a similar loss of viability (1.27% and 5.3% for CON and E7 cells respectively) although the protective effect of dUTPase was still evident.

### Effect of ZD9331 on DNA integrity

### *Nascent DNA*

A 4 h exposure to ZD9331 (1 µM) caused no measurable effect on nascent DNA in A549 cells but by 24 h substantial DNA damage was measured (Table 1). In contrast, MOR cells showed an earlier and greater amount of damage to nascent DNA. HX147 cells showed a similar pattern of DNA damage in response to ZD9331 as A549 cells.

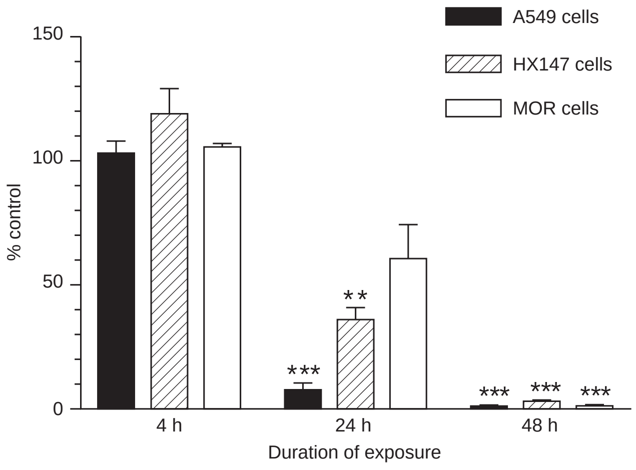

**Figure 2** Effect of ZD9331 on colony formation in 3 human lung tumour cell lines. Cells were treated with 1 µM ZD9331 for the indicated times before plating out in DFM. The data represents the results of 3 experiments carried out in duplicate. Bars represent SD.*** and ** denotes a significant ($P < 0.0001$ and $P < 0.01$ respectively) loss of viability

**Table 1** Nascent and mature DNA damage rad equivalents.

| | A549 | MOR | HX147 |
|---|---|---|---|
| **Nascent DNA damage** | | | |
| 4 h | 0 | 117 ± 5.4 | 0 |
| 24 h | 264 ± 9.8 | 500: 608 | 134 ± 153.4 |
| **Mature DNA damage** | | | |
| 4 h | 0 | 0 | ND |
| 24 h | 471 ± 95 | 134 ± 28 | 153 ± 21 |
| 48 h | 268: 279 | 281: 295 | ND |

Data represents the mean of 2–3 experiments conducted in duplicate (± SD). ND=not determined.

### Mature DNA

In A549 cells no damage to mature DNA was measured following a 4 h exposure to ZD9331. However by 24 h, damage to mature DNA was substantial (471 ± 95 rad equivalents) (Table 1). In contrast 3–4-fold less damage was observed in MOR cells following a 24 h exposure to ZD9331. A 48 h exposure to drug resulted in a similar extent of damage to mature DNA in A549 and MOR cells. HX147 cells had less mature DNA damage compared to A549 cells.

### Uracil DNA glycosylase (UDG) activity

A549 cells had significantly ($P > 0.05$) higher UDG activity (932 ± 248 pmole uracil $min^{-1}$ $mg^{-1}$) compared to MOR and HX147 cells (201 ± 30.6 and 166.9 ± 16.4 pmole uracil $min^{-1}$ $mg^{-1}$ respectively) (Figure 4). UDG activities in CON and dutE7 were not significantly different (404 ± 88 and 454 ± 238 pmole uracil $min^{-1}$ $mg^{-1}$ respectively).

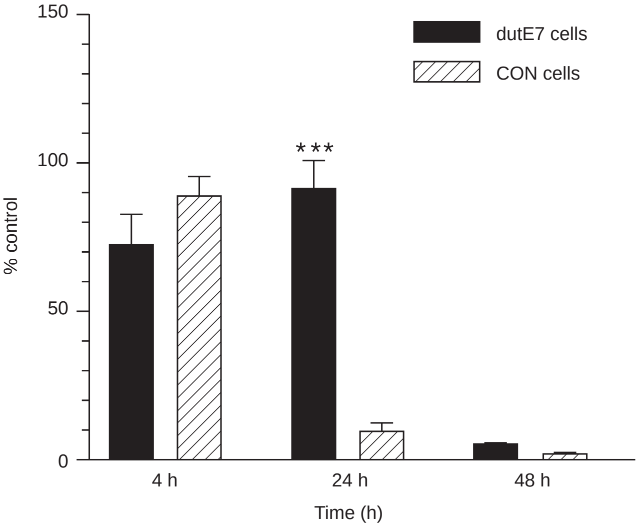

**Figure 3** Effect of ZD9331 on colony formation in HT29 dutE7 and CON cell lines. Cells were treated with 1 µM ZD9331 for the indicated times before plating out in DFM. The data represents the results of 3 experiments carried out in duplicate. Bars represent SD. *** and ** denotes a significant ($P < 0.0001$ and $P < 0.01$ respectively) loss of viability

*© 2001 Cancer Research Campaign*

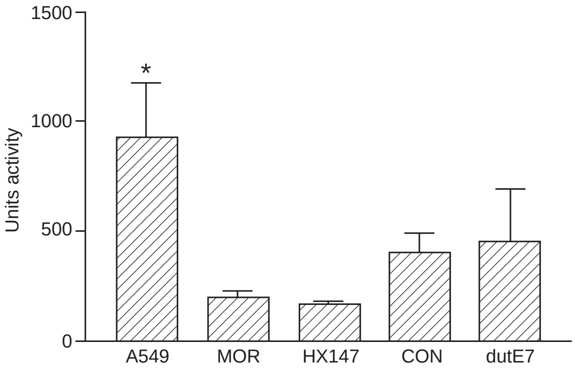

**Figure 4** UDG activity. Cells in exponential growth were harvested and assayed for UDG activity as detailed in Materials and Methods. UDG activity is expressed as pmole uracil released $min^{-1}$ $mg^{-1}$ protein. The data represents the results of 3–4 experiments carried out in duplicate. Bars represent SD. * denotes a significantly ($P < 0.05$) higher UDG activity in A549 cells than MOR and HX147 cells

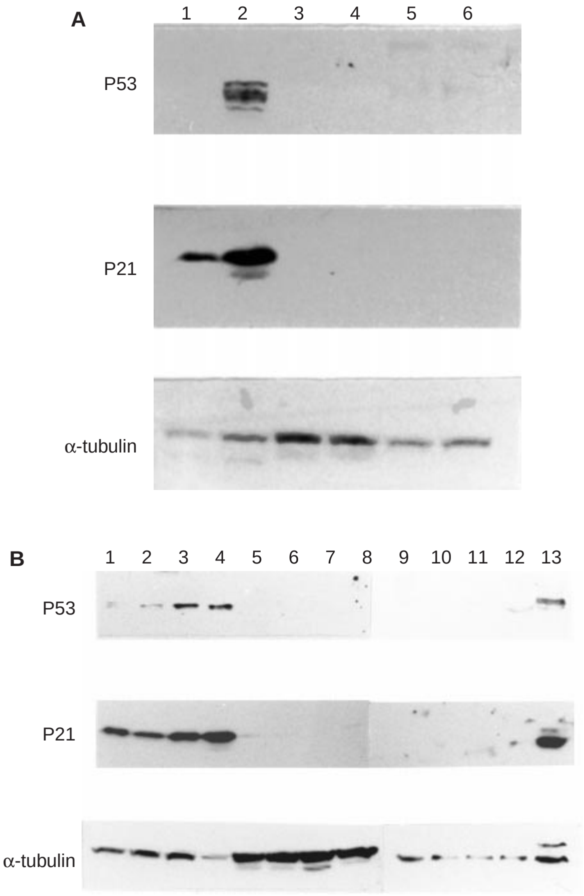

**Figure 5** Changes in the level of p53 and p21 following (**A**) irradiation and (**B**) 1 μM ZD9331. (**A**) Lanes: (1) A549 control; (2) A549 5 Gy; (3) MOR control; (4) MOR 5 Gy; (5) HX147 control; (6) HX147 5 Gy. (**B**) Lanes: (1) A549 control; (2) A549 4 h; (3) A549 24 h; (4) A549 48 h; (5) MOR control; (6) MOR 4 h; (7) MOR 24 h: (8) MOR 48 h; (9) HX147 control; (10) HX147 4 h; (11) HX147 24 h; (12) HX147 48 h; (13) 5 Gy A549 positive control. Tubulin was used to confirm equal protein loading

### Changes in the expression of p53 and p21 following exposure to ZD9331

Using Western blot analysis, levels of p53 were undetectable in all 3 untreated human tumour cell lines. The functional p53 status in these cell lines was confirmed by irradiation. Following irradiation (5 Gy), only A549 cells showed increased p53 expression. Low expression of p21 protein was detected in A549 control cells and was markedly elevated following irradiation. However, p21 was not detectable in either MOR or HX147 before or after irradiation (Figure 5A). Treatment with ZD9331 caused a time-related increase in p53 protein in A549 cells; p53 was clearly detected by 24 h and rose considerably by 48 h (Figure 5B). Expression of p21 was also increased in response to ZD9331. These proteins were not detected in MOR or HX147 cells following ZD9331 exposure.

### DISCUSSION

The main aim of this study was to further investigate the relative roles of dTTP and uracil misincorporation in cellular sensitivity to TS inhibition. 3 human tumour cell lines and a pair of isogenic cell lines differing only in the expression of dUTPase have been used. Against a background of dTTP depletion (>80% compared to untreated controls), A549, HX147 and MOR cells accumulate markedly different amounts of dUTP (57, 17 and 1.2 pmole/$10^6$ cells respectively) following a 24 h exposure to 1 μM ZD9331 (Webley et al, 2000). A longer duration of exposure to the drug served to further expand the dUTP pools in A549 and HX147 cells but had little effect on MOR cells. This difference in dUTP accumulation broadly related to dUTPase activity and protein expression. The cell lines also displayed differences in sensitivity to the growth inhibitory effects of ZD9331 depending on the duration of exposure; A549 cells were equally sensitive to a short and long exposure, whereas MOR and HX147 cells were more sensitive to a long drug exposure. The current study has addressed whether the extent of dUTP accumulated is related to changes in the cell cycle, loss of viability and DNA damage following treatment with ZD9331. In addition, the roles of p53 and p21 in mediating cell cycle and cytotoxic events following TS inhibition have also been addressed.

The viability of the cell lines after ZD9331 exposure depended on the duration of exposure; A549 cells showed an earlier (24 h) cytotoxic response compared to MOR cells which did not significantly lose clonogenicity until 48 h. ZD9331 was less cytotoxic to HX147 cells than A549 cells. A direct association therefore exists between the amount of dUTP formed and the time required to significantly lose viability. This observation is similar to that previously reported with fluoropyrimidines (Canman et al, 1993). Following exposure to FdUrd, loss of viability was later (24–32 h) in SW620 cells which accumulated low levels of dUTP than in HT29 cells (10–16 h) that accumulated 45-fold more dUTP. The neotransfected HT29 cells (CON) (mutant p53) accumulate a similar level of dUTP as A549 cells (wild-type p53) following exposure to ZD9331 (Brown et al, 1997). The former also showed an early loss in clonogenicity compared to the dUTPase transfected dutE7 HT29 cell line although this difference was lost at later time points. This observation has been reported with other TS inhibitors (Canman et al, 1994; Parsels et al, 1998). In addition, a greater difference in the level of growth inhibition was observed between CON and dutE7 at 24 h (71 fold), than at 120 h (3.8 fold). Therefore, against the same genetic background, reduced dUTP pools (and presumably DNA damage) can decrease the cytotoxic response to TS inhibition but this effect is largely time dependent.

 © 2001 Cancer Research Campaign

Alterations in the cell cycle in response to antifolates such as methotrexate (Taylor et al, 1982; Savage and Prasad, 1988) RTX (Matsui et al, 1996; Tonkinson et al, 1997) and other TS inhibitors (Skelton et al, 1998) have previously been reported. The cell cycle effects of TS inhibition (S-phase arrest resulting in reduced numbers of cells in G2/M and increased numbers of cells in G1) appears to be a universal consequence of TS inhibition and also occur following ZD9331 exposure. The cell cycle pattern typical of TS inhibition was observed by 4 h following drug treatment in all the cell lines studied. Thus in contrast to cytotoxicity, cell cycle arrest does not appear to depend on variations in dUTP accumulation (or as discussed later, p53 function). Also, the differential cytotoxicity observed is occurring during a cell cycle arrested state.

Re-suspension of ZD9331-treated cells (HT29 CON and dutE7) in drug-free medium restored normal S phase progression for up to 24 h at least in some cells. Thus arrest at the G1/S border may be a consequence of incomplete DNA synthesis due to reduced dTTP pools and not the irreversible inactivation of the DNA synthesis machinery. The RTX induced S-phase arrest in HCT-8 and SW620 cells was also shown to be due to the block in DNA synthesis as result of dTTP depletion (Matsui et al, 1996) and the addition of exogenous thymidine to the arrested cells restored normal S-phase progress.

The formation of DNA single strand breaks has widely been reported following treatment with TS inhibitors (Curtin et al, 1991; Yin et al, 1992; Canman et al, 1993). Nascent DNA damage in response to ZD9331 was observed in MOR cells as early as 4 h and by 24 h a significant extent of damage was measured. In contrast, A549 cells were less sensitive to this type of damage. However, cytotoxicity in MOR cells was significantly less than in A549 cells although it is thought that the inhibition of nascent DNA synthesis for an extended period of time would eventually result in commitment to cell death (Houghton et al, 1994).

It is known that cells in S-phase are most sensitive to the effects of TS inhibitors (Tang et al, 1996) and it is not clear why a larger degree of nascent DNA damage was not found in the A549 cell line. More damage of this nature may have been expected since dTTP pools were depleted (> 80%) to the same extent as in the other cell lines (Webley et al, 2000), and because of the large accumulation of dUTP that occurs following ZD9331 treatment. Damage to mature DNA is believed to reflect the inability to repair spontaneously occurring damage and in this context, may reflect the effect of the misincorporation process.

The presence of mature DNA single strand breaks is consistent with the corresponding loss of viability following treatment with ZD9331. Previous work found that the extent of mature DNA damage in HT29 and SW620 was proportional to the intracellular dUTP accumulated in these cells following FdUrd exposure (Canman et al, 1993). The extent of dUTP accumulation has also been shown to correlate with the extent of DNA damage (Curtin et al, 1991). It has been demonstrated that by transfecting HT29 cells with the antisense-expressing construct of the DUT-N open reading frame, dUTP accumulation and DNA damage can be increased (Ladner and Caradonna, 1997). As expected from the large difference in dUTP accumulation, A549 cells had higher levels of mature DNA damage than MOR and HX147 cells. UDG is a repair enzyme that rapidly removes misincorporated uracil from DNA creating alkaline labile apyrimidinic sites. The significantly ($P < 0.05$) higher UDG activity in A549 cells compared to HX147 cells, may have also contributed to the formation of mature DNA damage.

The similarity between the 24 and 120 h $IC_{50}$ concentrations for ZD9331 in A549 cells (Webley et al, 2000) suggests that these cells are largely committed to cell death within 24 h of drug exposure. This may be due to dUTP accumulation and the level of mature DNA damage that occur during the first 24 h exposure to ZD9331. Following a 48 h exposure to ZD9331 DNA damage was reduced, presumably because of cell loss due to apoptosis. MOR cells required a longer period of exposure to ZD9331 to show significant mature DNA strand breaks (compared to A459 cells). Other studies, using cells that do not accumulate dUTP during a thymineless state, have noted that the appearance of significant damage to mature DNA is a late event occurring between 24 and 48 h (Houghton et al, 1994; Sundseth et al, 1997).

Exposure of A549, MOR and HX147 cell lines to radiation confirmed that only the A549 cells have functional p53 and it was therefore not surprising that only A549 cells showed an elevation in p53 following ZD9331. However, all cell lines displayed an obvious G1/S arrest after only a 4 h exposure to ZD9331. In addition, this arrest occurred before the large rise in p53 protein expression in A549 cells. The same cell cycle effects following ZD9331 were observed in the p53-mutant HT29 dUTPase transfected cell line (dutE7) and its neotransfected control. p53 has been reported to play a passive role in RTX-induced cell cycle perturbation in HCT-8 (wt53) and SW480 (mutant p53) (Matsui et al, 1996).

Cellular response to TS inhibitors is complex but is probably mediated through a combination of both dTTP depletion and uracil misincorporation. It is difficult to evaluate the relative importance of these concurrent effects as dUTP accumulation will occur (to different extents) only in the presence of dTTP depletion. The expression of enzymes such as dUTPase, repair enzymes such as UDG and downstream factors (Fisher et al, 1993) are also important factors. Nevertheless, these studies have confirmed that the cell cycle events that are typical of TS inhibition occur regardless of p53 function and are independent of dUTP accumulation. However, there is an association between *early* cytotoxicity to TS inhibitors and the extent of dUTP accumulation. Importantly, in some cell lines, (e.g. MOR) the mechanism of cell death following TS inhibition is dUTP independent confirming the importance of dTTP depletion and other dNTP perturbations to the process of eventual cell death. It could be argued that the early loss of viability observed in the high dUTP accumulating A549 cell line, is due to the presence of functional p53 rather than uracil misincorporation per se. However, in the p53 mutant HT29 cell line high accumulation of dUTP appears to confer early cytotoxicity compared to a dUTPase expressing transfect. The use of paired cell lines with known p53 function (e.g. transfected with p53 inducible constructs or p53 'knockouts') would provide further insight into the relative roles of dUTPase and p53 in promoting cell death in TS inhibited cells. In addition, high UDG activity in cell lines that accumulate dUTP may exacerbate the effects of TS inhibitors thereby promoting DNA damage. The therapeutic importance of dUTPase and UDG expression in relation to p53 status remains to be determined and further studies using appropriately transfected (dUTPase and UDG) cell lines are warranted.

### ACKNOWLEDGEMENTS

This work was supported by the Cancer Research Campaign (Programme grant SP2330/0201 and PhD Studentship).

© 2001 Cancer Research Campaign

### REFERENCES

Aherne GW, Hardcastle A, Raynaud F and Jackman AL (1996) Immunoreactive dUMP and TTP pools as an index of thymidylate synthase inhibition; Effect of Tomudex (ZD1694) and a nonpolyglutamated quinazoline antifolate (CB30900) in L1210 mouse leukaemia cells. *Biochem Pharmacol* **51**: 1293–1301

Aherne GW and Brown SD (1999) The role of uracil misincorporation in thymineless death. In *Anticancer Drug Development Guide: Antifolate Drugs in Cancer Therapy*. Ed., AL Jackman. Humana Press Inc., Totowa, NJ. pp 409–421

Arrendondo MA, Yin MB, Lu K, Schoeber C, Slocum HK and Rustum YM (1994) Decreased c-myc protein accompanied by increased p53 and Rb is associated with thymidylate synthase inhibition and cell cycle retardation induced by ZD1694. *Proc Am Assoc Cancer Res* **35**: 304

Brown SD, Hardcastle A and Aherne GW (1997) Deoxyuridine triphosphatase (dUTPase) expression and cellular response to TS inhibitors. In: *Chemistry and Biology of Pteridines and Folates*. Pfleider W and Rokos H (eds), Blackwell Science, Berlin, pp 271–274

Brynolf K, Eliasson R and Reichard P (1978). Formation of Okazaki fragments in polyoma DNA synthesis caused by misincorporation of uracil. *Cell* **13**: 573–580

Canman CE, Lawrence TS, Shewach DS, Tang HY and Maybaum J (1993) Resistance to fluorodeoxyuridine induced DNA damage and cytotoxicity correlates with an elevation of deoxyuridine triphosphatase activity and failure to accumulate deoxyuridine triphosphate. *Cancer Res* **53**: 1–6

Canman CE, Radany EH, Parsels LA, Davis MA, Lawrence TS and Maybaum J (1994) Induction of resistance to fluorodeoxyuridine cytotoxicity and DNA damage in human tumour cells by expression of *Escherichia-coli* deoxyuridine triphosphatase. *Cancer Res* **54**: 2296–2298

Caradonna SJ and Cheng Y (1980) Uracil DNA-glycosylase: purification and properties of this enzyme isolated from blast cells of acute myelocytic leukemia patients. *J Biol Chem* **255**: 2293–2300

Chong L and Tattersall MHN (1995) 5, 10 Dideazatetrahydrofolic acid reduces toxicity and deoyadenosine triphosphate pool expansion in cultural L1210 cells treated with inhibitors of thymidylate synthase. *Biochem Pharmacol* **49**: 819–827

Curtin NJ, Harris AL and Aherne GW (1991) Mechanisms of cell death following thymidylate synthase inhibition: 2 deoxyuridine 5 triphosphate accumulation, DNA damage, and growth inhibition following exposure to CB3717 and dipyridamole. *Cancer Res* **51**: 2346–2352

Darzynkiewicz Z, Bruno S, Del Bino G, Gorczyca W, Hotz MA, Lassota P and Traganos F (1992) Features of apoptotic cells measured by flow cytometry. *Cytometry* **13**: 795–808

Fisher TC, Milner AE, Gregory CD, Jackman AL, Aherne GW, Hartley JA, Dive C and Hickman J (1993) Bcl-2 modulation of apoptosis induced by anticancer drugs-resistance to thymidylate stress is independent of classical resistance pathways. *Cancer Res* **53**: 3321–3326

Goulian M, Bleile BM, Dickey LM, Grafstrom RH, Ingraham HA, Neynaben SA, Peterson MS and Tseng BY (1986) Mechanism of thymineless death. *Adv Exp Med Biol* **195**: 89–95

Hartwell L and Kastan MB (1994) Cell cycle control and cancer. *Science (Washington DC)* **266**: 1821–1828

Houghton JA, Harwood FG and Houghton PS (1994) Cell cycle control processes determine cytostasis or cytotoxicity in thymineless death of colon cancer cells. *Cancer Res* **54**: 4967–4973

Houghton JA, Tilman DM and Harwood FG (1995) Ratio of 2′-deoxyadenosine-5′-triphosphate/thymidine-5′-triphosphate influences the commitment of human colon carcinoma cells to thymineless death. *Clin Cancer Res* **1**: 723–730

Jackman AL and Judson IR (1996) The new generation of thymidylate synthase inhibitors in clinical study. *Exp Opin Invest Drugs* **5**: 719–736

Jackman AL, Kimbell R, Aherne GW, Brunton L, Jansen G, Stephens TC, Smith MC, Wardleworth JM and Boyle FT (1997) Cellular pharmacology and invivo activity of a new anticancer agent ZD9331: A water-soluble, nonpolyglutamatable, quinazoline inhibitor of thymidylate synthase. *Clin Cancer Res* **3**: 911–921

Jackson RC, Jackman AL and Calvert AH (1983) Biochemical effects of quinazoline inhibitor of thymidylate synthase CB3717 on human lymphoblastoid cells. *Biochem Pharmocol* **33**: 3783–3790

Kohn KW (1981) Principles and practice of DNA filter elution. *Pharmac Ther* **49**: 55–77

Ladner R and Caradonna S (1997) Lowering dUTPase levels through antisense induces sensitivity to fluorodeoxyuridine in HT29 cells. *Proc Am Assoc Cancer Res* **38**: 614

Matsui S, Arredondo MA, Wrzosek C and Rustum YM (1996) DNA damage and p53 induction do not cause ZD1694-induced cell cycle arrest in human colon carcinoma cells. *Cancer Res* **56**: 4715–1723

Muller-Weeks S and Caradonna S (1996) Specific association of cyclin like uracil-DNA glycosylase with the proliferating cell nuclear antigen. *Exp Cell Res* **226**: 346–355

Nelson WG and Kastan MB (1994) DNA strand breaks: the DNA template alterations that trigger p53-dependent DNA damage response pathways. *Mol Cell Biol* **14**: 1815–1823

O'Connor PM (1987) Mammalian G1 and G2 phase checkpoints. *Cancer Surv* **29**: 151–182

Parsels LA, Parsels JD, Wagner LM, Loney TL, Radany EH and Maybaum J (1998) Mechanism and pharmacological specificity of dUTPase-mediated protection from DNA damage and cytotoxicity in human tumor cells. *Cancer Chemother Pharmacol* **42**: 357–362

Savage JRK and Prasad R (1988) Generalized blocking in S phase by methotrexate. *Mut Res* **201**: 195–201

Skelton LA, Ormerod MG, Titley JC and Jackman AL (1998) Cell cycle effects of CB30865, a lipophilic quinazoline-based analogue ICI 198583 with an undefined mechanism of action. *Cytometry* **33**: 56–66

Sundseth R, Singer S, Yates B, Smith G, Ferone R and Dev I (1997) Thymineless apoptotic death induced by 1843U89 in human tumour cell lines is independent of dUTP accumulation and misincorporation of dUMP into DNA. *Proc Am Assoc Cancer Res* **38**: 476

Tang HY, Weber KL, Lawrence TS, Merchant AK and Maybaum J (1996) Dependence of fluorodeoxyuridine-induced cytotoxicity and megabase DNA fragmentation on S phase progression in HT29 cells. *Cancer Chemother Pharmacol* **37**: 486–490

Taylor IW, Slowiaczek P, Francis PR and Tattersall MHN (1982) Purine modulation of methotrexate cytotoxicity in mammalian cell lines. *Cancer Res* **42**: 5159–5164

Tonkinson JL, Marder P, Andis SL, Schultz RM, Gossett LS, Shih C and Mendelsohn LG (1997) Cell cycle effects of antifolate antimetabolites: implications for cytotoxicity and cytotasis. *Cancer Chemother Pharmacol* **39**: 521–531

Webley SD, Hardcastle A, Ladner RD, Jackman AL and Aherne GW (2000) Deoxyuridine triphosphate (dUTPase) expression and sensitivity to the thymidylate synthase (TS) inhibitor ZD9331. *Br J Cancer* **83**: 792–799

Yin MB, Guimalaes MA, Zhang Z, Arrendondo MA and Rustum YM (1992) Time dependence of DNA lesions and growth inhibition by ICI D1694, a new quinazoline antifolate thymidylate synthase inhibitor. *Cancer Res* **52**: 5900–5905

Zambetti GP and Levine AJ (1993) A comparison of the biological activities of wild type and mutant p53. *FASEB J* **7**: 855–865

 *© 2001 Cancer Research Campaign*
